# Insightful Backbone Modifications Preventing Proteolytic Degradation of Neurotensin Analogs Improve NT*S1*-Induced Protective Hypothermia

**DOI:** 10.3389/fchem.2020.00406

**Published:** 2020-06-05

**Authors:** Santo Previti, Mélanie Vivancos, Emmanuelle Rémond, Sabrina Beaulieu, Jean-Michel Longpré, Steven Ballet, Philippe Sarret, Florine Cavelier

**Affiliations:** ^1^Institut des Biomolécules Max Mousseron, IBMM, UMR-5247, CNRS, Université de Montpellier, ENSCM, Montpellier, France; ^2^Departments of Bioengineering Sciences and Chemistry, Research Group of Organic Chemistry, Vrije Universiteit Brussel, Brussels, Belgium; ^3^Department of Pharmacology-Physiology, Faculty of Medicine and Health Sciences, Institut de Pharmacologie de Sherbrooke, Université de Sherbrooke, Sherbrooke, QC, Canada

**Keywords:** NTS1, reduced peptide bonds, unnatural amino acids, proteolytic stability, hypothermia

## Abstract

Therapeutic hypothermia represents a brain-protective strategy for multiple emergency situations, such as stroke or traumatic injury. Neurotensin (NT), which exerts its effects through activation of two G protein-coupled receptors, namely NTS1 and NTS2, induces a strong and long-lasting decrease in core body temperature after its central administration. Growing evidence demonstrates that NTS1 is the receptor subtype mediating the hypothermic action of NT. As such, potent NTS1 agonists designed on the basis of the minimal C-terminal NT(8-13) bioactive fragment have been shown to produce mild hypothermia and exert neuroprotective effects under various clinically relevant conditions. The high susceptibility of NT(8-13) to protease degradation (half-life <2 min) represents, however, a serious limitation for its use in pharmacological therapy. In light of this, we report here a structure-activity relationship study in which pairs of NT(8-13) analogs have been developed, based on the incorporation of a reduced Lys^8^-Lys^9^ bond. To further stabilize the peptide bonds, a panel of backbone modifications was also inserted along the peptide sequence, including Sip^10^, D-Trp^11^, Dmt^11^, Tle^12^, and TMSAla^13^. Our results revealed that the combination of appropriate chemical modifications leads to compounds exhibiting improved resistance to proteolytic cleavages (>24 h; **16**). Among them, the NT(8-13) analogs harboring the reduced amine bond combined with the unnatural amino acids TMSAla^13^ (**4**) and Sip^10^ (**6**) or the di-substitution Lys^11^ - TMSAla^13^ (**12**), D-Trp^11^-TMSAla^13^ (**14**), and Dmt^11^-Tle^12^ (**16**) produced sustained hypothermic effects (−3°C for at least 1 h). Importantly, we observed that hypothermia was mainly driven by the increased stability of the NT(8-13) derivatives, instead of the high binding-affinity at NTS1. Altogether, these results reveal the importance of the reduced amine bond in optimizing the metabolic properties of the NT(8-13) peptide and support the development of stable NTS1 agonists as first drug candidate in neuroprotective hypothermia.

## Introduction

Mild hypothermia (32–35°C) has been proven to exert neuroprotective effects in a variety of neurological conditions, such as global ischemia after cardiac arrest, hypoxic-ischemic encephalopathy, ischemic stroke, and traumatic injury (Huber et al., 2019). Indeed, therapeutic cooling has been described to counteract many of the deleterious processes occurring in the setting of cerebral ischemia, including neuroinflammation, free radical production, excitotoxicity, and apoptosis, as well as blood–brain barrier disruption (Sun et al., 2019). To date, hypothermia is achieved by internal or external cooling interventions (Chen et al., 2014). However, these physical methods have serious limitations, which include slow onset of action, undesirable shivering and vasoconstriction responses, need for general anesthesia, and poor ability to implement in an out-of-hospital environment (Sun et al., 2019). There is, therefore, a growing interest to develop drugs that safely reduce the body temperature by controlling the hypothalamic set point (Kurisu et al., 2019).

Neurotensin (NT) is an endogenous tridecapeptide (pGlu–Leu–Tyr–Glu–Asn–Lys–Pro–Arg–Arg–Pro–Tyr–Ile–Leu–OH) first isolated in 1973 from the bovine hypothalamus (Carraway and Leeman, 1973) that acts as an active neuromodulator/neurotransmitter within the central nervous system (Boules et al., 2013). Among the first NT-induced effects to be reported was its ability to produce a marked and sustained hypothermia after intracisternal or intracerebroventricular injection in a variety of mammals, including rat, mouse, and monkey (Bissette et al., 1976; Nemeroff et al., 1977; Fantegrossi et al., 2005). Accordingly, intracerebral injection of NT in regions known to be involved in thermoregulatory homeostasis and rich in NT innervation, such as the anterior hypothalamus and medial preoptic area, produces a dose-dependent decrease in body temperature (Martin et al., 1980; Bissette et al., 1982; Kalivas et al., 1985). In addition to its role in the neural control of thermoregulation, central delivery of NT, and derivatives also displays potent analgesia and antipsychotic-like effects (Dobner, 2006; St-Gelais et al., 2006; Feng et al., 2015).

Brain NT exerts its effects through binding and activation of three different receptors: NTS1 and NTS2, both belonging to the class A G protein-coupled receptor (GPCR) family, and NTS3, a sortilin-like receptor, characterized by a single transmembrane domain (Vincent et al., 1999; Sarret and Cavelier, 2018). There is now compelling evidence to support that NTS1 is the receptor responsible for the hypothermic effects of NT agonists. Indeed, highly potent NTS1 agonists, such as the NT69L, PD149163, and NT-2 Eisai peptides produce a long-lasting hypothermia following peripheral administration (Tyler-McMahon et al., 2000; Katz et al., 2001; Feifel et al., 2010). In addition, *in vivo* blockade of NTS1 receptor expression, using antisense strategies or mice lacking NTS1, provides direct evidence for the relationship between hypothermia and NTS1 binding (Tyler-McMahon et al., 2000; Pettibone et al., 2002; Remaury et al., 2002; Mechanic et al., 2009). Likewise, the use of NTS2-selective analogs, such as JMV431 or NT79, and inactivation of NTS2 further confirm the main role played by NTS1 in brain and body temperature control (Dubuc et al., 1999; Boules et al., 2010). More recently, a series of studies has highlighted the benefit of achieving regulated reduction of body temperature using NTS1 agonists in different clinically relevant situations. For instance, systemic administration of NT69L, PD149163, or HPI-201 (formally ABS-201) produced mild hypothermia and exerted neuroprotective effects after ischemic stroke, intracerebral hemorrhage, resuscitation from cardiac arrest, and traumatic brain injury (Katz et al., 2001; Choi et al., 2012; Wei et al., 2013; Gu et al., 2015; Lee et al., 2016; Xue et al., 2017; Zhao et al., 2020; Zhong et al., 2020).

**Chart 1:**
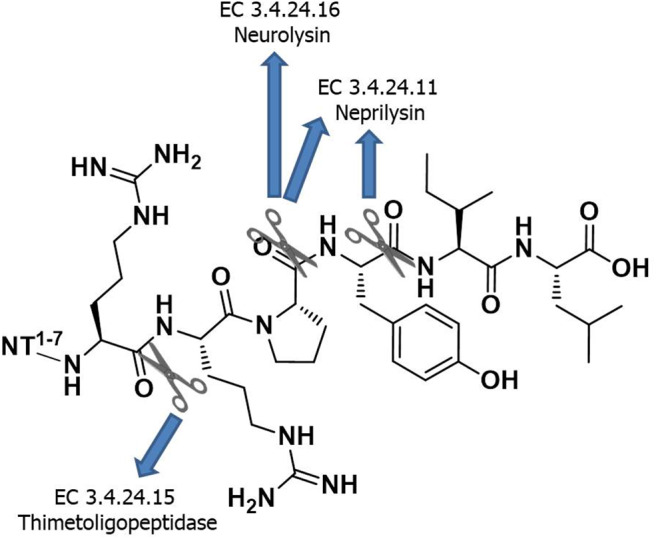
Minimum sequence of NT required for the biological activity and its enzymatic degradation sites.

Following NT's isolation, multiple structure–activity relationship (SAR) studies have enabled the identification of the *C*-terminal peptide fragment H-Arg-Arg-Pro-Tyr-Ile-Leu-OH [i.e., NT(8-13)], as the minimum sequence for producing NT activity (Uhl et al., 1977; St-Pierre et al., 1981; Granier et al., 1982). Of note, the positive charges at Arg^8^ and Arg^9^ as well as the side chain length at position 9 are important for retaining adequate NTS1 binding affinity (Cusack et al., 1995; Hadden et al., 2005). The main drawback in the use of NT(8-13) as a drug is its extremely short biological half-life (<2 min) due to rapid *in vivo* proteolysis by several peptidases (Chart 1). After intravenous infusion, NT(8-13) is indeed degraded by a combination of three metalloendopeptidases, referred to as EC 3.4.24.11 (also known as nephrilysin), EC 3.4.24.15 (thimetoligopeptidase), and EC 3.4.24.16 (neurolysin), that show cleaving activity at the Arg^8^-Arg^9^, Pro^10^-Tyr^11^, and Tyr^11^-Ile^12^ positions (Checler et al., 1984, 1985, 1986). In the last two decades, many efforts were dedicated to increase NT's half-life, without affecting the biological activity (Sarret and Cavelier, 2018). Among the strategies used, modifications such as reduced peptide bonds, *N*-terminal methylation and acetylation, cyclization, modifications to the peptide backbone, and incorporation of unnatural amino acids represent the classical approaches used by medicinal/peptide chemists to improve the peptide biostability (Adessi and Soto, 2002; Werner et al., 2016).

**Chart 2:**
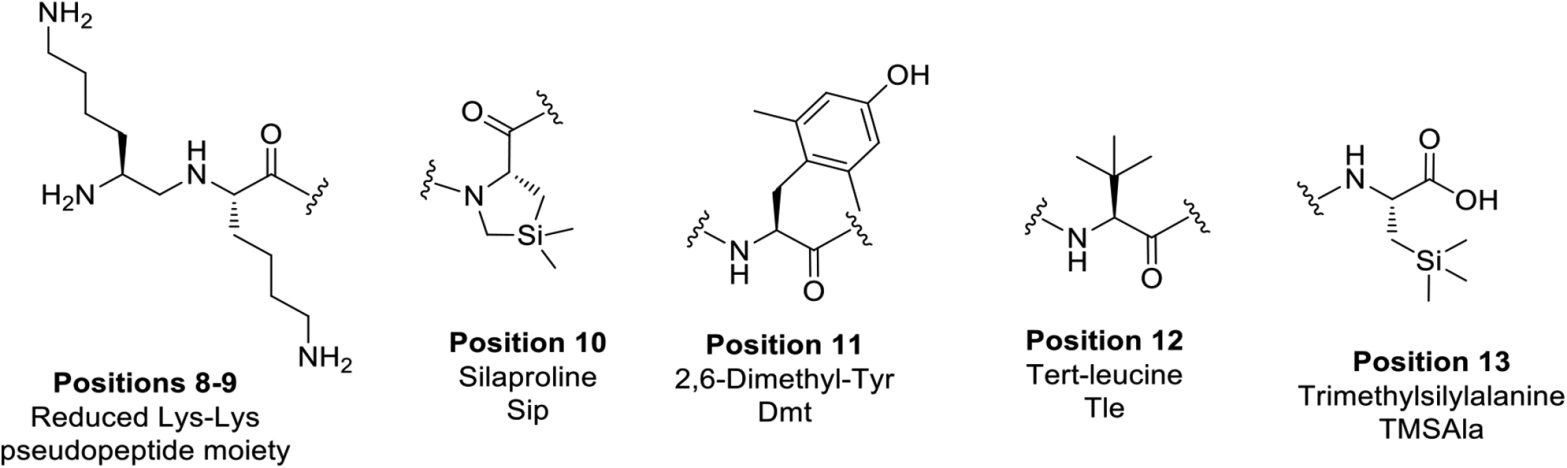
Unnatural amino acids and backbone modifications inserted into the NT(8-13) analogs.

In view of designing proteolytically stable NT(8-13) analogs, we decided here to synthesize a series of eight specific pairs of NT(8-13) derivatives, divided in two groups based on the incorporation of a reduced Lys^8^-Lys^9^ pseudopeptide bond. Additional backbone modifications were also introduced at Pro^10^, Tyr^11^, Ile^12^, and Leu^13^ (Chart 2). We then studied how these chemical substitutions influenced the peptide plasma stability, NTS1/NTS2 binding affinity as well as their ability to induce changes in body temperature.

## Materials and Methods

### Chemistry

All data regarding chemistry section are reported in the Supporting Information. Please note that among the NT(8-13) analogs described here, some of them were already described in previous publications, as indicated in the appropriate section (see Table S1).

### Biology

#### Competitive Radioligand Binding Assay

CHO-K1 cells stably expressing hNTS1 (ES-690-C from PerkinElmer) or 1321N1 cells stably expressing hNTS2 (ES-691-C from PerkinElmer) were cultured, respectively, in DMEM/F12 or DMEM. Culture media were supplemented with 10% FBS, 100 U/mL penicillin, 100 μg/mL streptomycin, 20 mM HEPES, and 0.4 mg/mL G418, and cells were incubated at 37°C in a humidified chamber at 5% CO_2_. All media and additives are from Wisent (St-Bruno, QC). Competitive radioligand binding experiments were performed by incubating 50 μg of freshly prepared cell membranes, expressing either hNTS1 or hNTS2, with 50 pM (for hNTS1) or 280 pM (for hNTS2) ^125^I-Tyr^3^-NT (2200 Ci/mmol, from PerkinElmer, Billerica, MA). Increasing concentrations diluted in binding buffer (50 mM Tris-HCl, pH 7.5, 0.2 % BSA) and ranging from 10^−11^ or 10^−10^ to 10^−5^ or 10^−4^ M of NT analogs were added. After 1 h of incubation at room temperature, the binding reaction mixture was transferred in polyethylenimine-coated 96-well-filter plates (Millipore, Billerica, MA). Reaction was terminated by filtration, and plates were washed three times with 200 μl of ice-cold binding buffer. Glass fiber filters were then counted in a γ-counter (1470 Wizard2, PerkinElmer). Non-specific binding was measured in the presence of 10^−5^ M unlabeled NT(8-13) and represented <5% of total binding. IC_50_ values were determined from the competition curves as the unlabeled ligand concentration inhibiting half of the ^125^I-Tyr^3^-NT-specific binding. Data were plotted using GraphPad Prism 8 using the One-site—Fit log (IC_50_) and represent the mean ± SEM of at least three separate experiments performed in triplicate.

IC_50_ calculated from the competitive radioligand binding assays were then transformed into *K*_i_ using the Cheng–Prusoff equation (Cheng and Prusoff, 1973):

$$\begin{array}{l} \text{Ki}=\frac{\text{I}{\text{C}}_{50}}{\left(\;1+\;\frac{\left[\text{L}\right]}{\text{Kd}}\;\right)} \end{array}$$

where L refers to the concentration of radiolabeled tracer (^125^I-[Tyr3]- NT) and K_d_ refers to the equilibrium dissociation constant of the radioligand. For NTS1, K_d_ = 0.7 nM, whereas for NTS2, K_d_ = 3.4 nM.

#### Plasma Stability

Rat plasma was obtained from blood by keeping the translucent phase after centrifugation at 15,000 g over 5 min. Plasma stability assay was carried out by incubating each compound at different incubation times in rat plasma at a final concentration of 0.156 mM. NT(8-13) and compounds without reduced amine bounds were incubated during short incubation times (0, 1, 2, 5, 10, and 30 min), whereas all analogs with reduced amine bounds, except compound **2**, were tested during longer incubation times (0, 1, 2, 4, 8, 16, and 24 h) at 37°C. Then, 70 μl of a solution containing 10% trichloroacetic acid (TCA) and 0.5% nicotinamine, an internal standard, was added to stop the degradation by proteases. After centrifugation at 15,000 g for 30 min, supernatant was filtered through 0.22-μm filter and analyzed by UPLC/MS (Water H Class Acquity UPLC, mounted with Acquity UPLC BEH C18 column, 1.7 μm, 2.1 × 50 mm and paired to a SQ Detector 2). Compounds **1**, **2**, and **5** were analyzed using the mass or UV spectrum. Quantification was done by determining the area under the curve (AUC) ratio of each compound over AUC of nicotinamine for each incubation time. Data were plotted into GraphPad Prism 8 and the half-life of each compound was calculated using one-phase decay fit and represented the mean ± SEM of minimum three separate experiments.

#### Animals, Housing, and Habituation

The experimental procedures in this study were approved by the Animal Care Committee of the Université de Sherbrooke (protocol n°035-18B) and were in accordance with policies and directives of the Canadian Council on Animal Care. Adult male Sprague-Dawley rats, weighing 175–225 g (Charles River laboratories, St-Constant, Québec, Canada), were maintained on a 12-h light/dark cycle with free access to food and water and were housed two per cage on Aspen shavings in a quiet room.

#### Intrathecal Injection

Rats were lightly anesthetized with isoflurane/oxygen (Baxter corporation, Mississauga, ON, Canada; 2 L/min) flow and 25 μl of each compound were injected intrathecally with a 27-G 1/2 needle (BD PrecisionGlide, USA) into the subarachnoid space between vertebrae L5 and L6. Analogs **1**–**14** were diluted in physiological saline at 5 mg/ml, whereas compounds **15** and **16** were diluted in DMSO. For body temperature measurement, the injected solution of these both compounds contained 6% DMSO at final concentration (30 μg/kg).

#### Body Temperature Measurement

Three consecutive days prior to testing, animals were individually acclimatized to manipulations and to a thermistor probe 5 min per day. The day of the test, experiments were always performed in a quiet room and between 8:00 and 12:00 PM. to reduce any variation related to the circadian rhythm. Body temperature was measured using a thermistor probe inserted into the rectum of adult Sprague-Dawley rats. Temperatures were recorded immediately before (baseline) and each 10 min for up to 60 min following intrathecal administration of saline, vehicle (6% DMSO) or NT analogs at 30 μg/kg. Changes in body temperature (Δ body temp) from baseline were determined for each animal. Data are expressed as mean ± SEM of 5 to 20 animals per condition.

### Statistical Analysis

Data are expressed as mean ± standard errors of the mean (SEM). All graphs and statistical analysis were performed using GraphPad Prism 8 (GraphPad software, La Jolla, CA, USA). A two-way ANOVA followed by Bonferroni's multiple comparisons test was used to determine significant differences between drug- and vehicle-treated rats at different timepoints post-injection.

## Results

### Design of the NT(8-13) Analogs

A series of eight specific pairs of NT(8-13) analogs was synthesized with the main objective to develop novel metabolically stable and potent NT(8-13) compounds (Table 1). Since a double Arg^8^-Arg^9^ substitution with Lys^8^-Lys^9^ is well-tolerated in terms of binding, this modification in positions 8–9 was preserved in all analogs (Uhl et al., 1977; St-Pierre et al., 1981; Granier et al., 1982). In addition, it has been previously shown that the incorporation of a reduced Lys^8^-Lys^9^ pseudopeptide bond in NT(8-13) analogs provides resistance to exonuclease cleavage with no significant influence on their biological activities (Lugrin et al., 1991; Fanelli et al., 2017). Thus, a second subset of NT(8-13) analogs encompassing a reduced amine bond between these two basic residues was prepared (Table 1). Introduction of reduced bonds is a well-known backbone modification, which induces a conformational change in the peptide, increases the flexibility in the peptide chain and also adds a positive charge into the backbone (Coy et al., 1988; Calbo et al., 1999). To further reduce the possible cleavage at the Pro^10^-Tyr^11^ and Tyr^11^-Ile^12^ scissile amine bonds, several residues were additionally substituted in the NT(8-13) sequence by unnatural amino acids based on previous findings. For example, compounds **3** (H-Lys-Lys-Pro-Tyr-Ile-TMSAla-OH) and **5** (H-Lys-Lys-Sip-Tyr-Ile-Leu-OH) carrying the silylated amino acids trimethylsilylalanine (TMSAla) and silaproline (Sip), respectively, showed an improved *K*_i_ value against NTS1 and a slightly improved plasma stability compared to NT(8-13) (Fanelli et al., 2015). For these reasons, the corresponding analogs bearing the reduced Lys-Lys pseudopeptide bond were synthesized, namely analogs **4** (H-LysΨ[CH_2_NH]-Lys-Pro-Tyr-Ile-TMSAla-OH) and **6** (H-LysΨ[CH_2_NH]-Lys-Sip-Tyr-Ile-TMSAla-OH). Additionally, we synthesized the NT(8-13) derivatives **7** (H-Lys-Lys-Sip-Tyr-Ile-TMSAla-OH) and **8** (H-LysΨ[CH_2_NH]Lys-Sip-Tyr-Ile-TMSAla-OH), in which both silylated amino acids were inserted.

**Table 1:** Chemical structures of NT analogs.

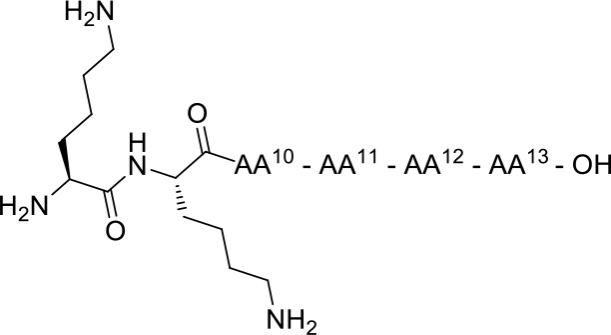			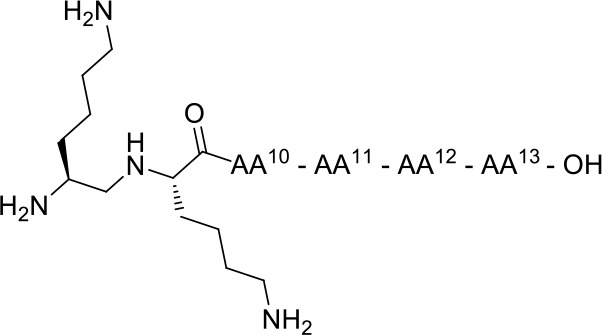	
1	H-Lys-Lys-Pro-Tyr-Ile-Leu-OH	2	H-$\text{Lys}\psi [{\text{CH}}_{2}\text{NH}]\text{Lys}$-Pro-Tyr-Ile-Leu-OH
3	H-Lys-Lys-Pro-Tyr-Ile- TMSAla-OH	4	H-$\text{Lys}\psi [{\text{CH}}_{2}\text{NH}]\text{Lys}$-Pro-Tyr-Ile- TMSAla-OH
5	H-Lys-Lys-Sip-Tyr-Ile-Leu-OH	6	H-$\text{Lys}\psi [{\text{CH}}_{2}\text{NH}]\text{Lys}$-Sip-Tyr-Ile-Leu-OH
7	H-Lys-Lys-Sip-Tyr-Ile- TMSAla-OH	8	H-$\text{Lys}\psi [{\text{CH}}_{2}\text{NH}]\text{Lys}$-Sip-Tyr-Ile-TMSAla-OH
9	H-Lys-Lys-Pro-Lys-Ile-Leu-OH	10	H-$\text{Lys}\psi [{\text{CH}}_{2}\text{NH}]\text{Lys}$-Pro-Lys-Ile-Leu-OH
11	H-Lys-Lys-Pro-Lys-Ile- TMSAla-OH	12	H-$\text{Lys}\psi [{\text{CH}}_{2}\text{NH}]\text{Lys}$-Pro-Lys-Ile-TMSAla-OH
13	H-Lys-Lys-Pro-(D)Trp-Ile- TMSAla-OH	14	H-$\text{Lys}\psi [{\text{CH}}_{2}\text{NH}]\text{Lys}$-Pro-(D)Trp-Ile-TMSAla-OH
15	H-Lys-Lys-Pro-Dmt-Tle-Leu-OH	16	H-$\text{Lys}\psi [{\text{CH}}_{2}\text{NH}]\text{Lys}$-Pro-Dmt-Tle-Leu-OH

*On the right, analogs bearing the reduced Lys^8^-Lys^9^ pseudopeptide bond. On the left, hexapeptides carrying the standard amide bond between Lys^8^ and Lys^9^. Colored residues indicate unnatural amino acids and backbone modifications*.

Previous studies have also revealed that substitution of the Tyr^11^ residue could provide a handle controlling NT receptor subtype selectivity (Dubuc et al., 1999; Boules et al., 2010; Einsiedel et al., 2011; Held et al., 2013; Fanelli et al., 2017). Accordingly, compound **9** (H-Lys-Lys-Pro-Lys-Ile-Leu-OH), previously reported in Richelson et al. (2008), Einsiedel et al. (2011), Fanelli et al. (2017), and Magafa et al. (2019), showed a significant selectivity in favor to NTS2 (NTS2/NTS1 ratio = 25). The corresponding analog **10** (H-LysΨ[CH_2_NH]Lys-Pro-Lys-Ile-Leu-OH) bearing the reduced Lys–Lys bond was now synthesized. In analogy with the first series of analogs, the high lipophilic TMSAla residue was introduced at the *C*-terminal end to replace the hydrophobic character of Leu^13^, giving rise to compounds **11** (H-Lys-Lys-Pro-Lys-Ile-TMSAla-OH) and **12** (H-LysΨ[CH_2_NH]Lys-Pro-Lys-Ile-TMSAla-OH).

The importance of the tyrosine residue at position 11 was further investigated by replacing Tyr^11^ by D-Trp^11^, which strongly increases the peptide's half-life as well as the selectivity toward NTS2 when compared to the native sequence (Jolicoeur et al., 1984; Richard et al., 2001). This substitution was combined with the incorporation of TMSAla at position 13 to afford compounds **13** (H-Lys-Lys-Pro-D-Trp-Ile-TMSAla-OH) and **14** (H-Lysψ[CH_2_NH]Lys-Pro-D-Trp-Ile-TMSAla-OH). Finally, we evaluated the plasma stability and its ability to regulate body temperature for **15** and **16**, which carry the unnatural amino acids 2′,6′-di-methyl-tyrosine (Dmt) and tert-leucine (Tle) in positions 11 and 12, respectively (**15**: H-Lys-Lys-Pro-Dmt-Tle-Leu-OH, **16**: H-Lysψ[CH_2_NH]Lys-Pro-Dmt-Tle-Leu-OH) (Eiselt et al., 2019).

### Peptide Synthesis

The synthesis of all NT(8-13) analogs are detailed in the Supporting Information. Please note that the synthesis of some of these NT(8-13) derivatives have already been published elsewhere: compounds **1**, **9**, and **11** in Lugrin et al. (1991) and Fanelli et al. (2017); compound **2** in Fanelli et al. (2017) and Lugrin et al. (1991); compounds **3** and **5** in Doulut et al. (1992), Vivet et al. (2000), René et al. (2013), and Fanelli et al. (2015); and compounds **15** and **16** in Fanelli et al. (2015) and Eiselt et al. (2019). Briefly, the designed hexapeptides were synthesized in solution starting from Boc-Leu-OMe commercially available, or Boc-TMSAla-OMe, which was previously described following the standard Boc strategy (Fanelli et al., 2015). Boc-Sip-OH and Boc-Lys(Boc)Ψ[CH_2_NH]Lys(Boc)-OH were synthesized, as previously reported (Doulut et al., 1992; Vivet et al., 2000; René et al., 2013; Fanelli et al., 2015).

### Biological Properties

#### Impact of The Peptide Backbone Modifications on Receptor Binding Affinity

**Figure 1:**
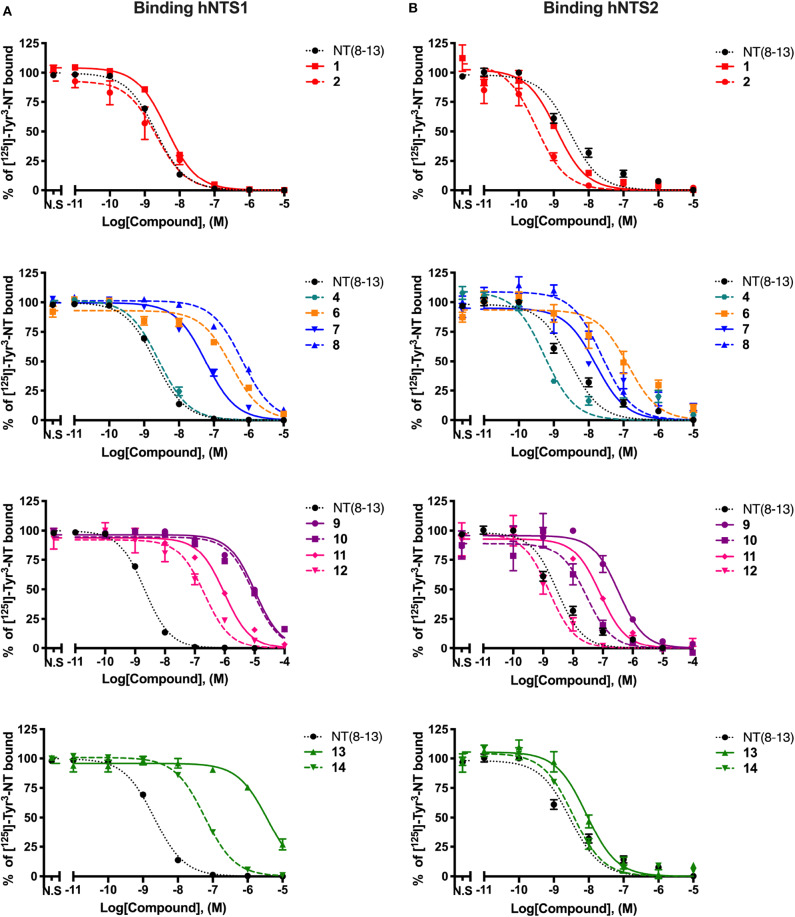
Displacement curves of ^125^I-[Tyr^3^]-NT by a series of NT(8-13) analogs at the NTS1 **(A)** and NTS2 **(B)** binding sites. Data are expressed ± SEM of at least three separate experiments. For compounds **3**, **5**, **15**, and **16**, *K*_i_ values were taken from the literature (Fanelli et al., 2015; Eiselt et al., 2019).

We first investigated the effects of adding a reduced Lys^8^-Lys^9^ pseudopeptide bond in combination with the substitution of Pro^10^, Tyr^11^, Ile^12^, and Leu^13^ by natural (Lys) or unnatural amino acids (Sip, TMSAla, D-Trp^11^, Dmt, Tle) on binding affinities for NTS1 and NTS2 receptors. To this aim, we determined the ability of these NT(8-13) derivatives to inhibit the binding of ^125^I-[Tyr^3^]-NT to membranes prepared from cells stably expressing either hNTS1 or hNTS2 receptors. The results are summarized in Table 2 and Figure 1. As indicated in Table 2, the radioligand binding studies on some NT(8-13) analogs, already reported in previous studies (Lugrin et al., 1991; Fanelli et al., 2015, 2017; Eiselt et al., 2019), were repeated in a same set of experiments for comparison purposes.

**Table 2:** Binding affinities toward the hNTS1 and hNTS2 receptors, plasma stability and body temperature of NT(8-13) and its derivatives.

**Name**	**Sequence**	**Binding**, ***K***_****i****_ **(nM)**		**NTS1/NTS2** **selectivity**	**Plasma stability** (**Half-life)**	**Hypothermia** **(30 μg/kg; i.t.)**
		**hNTS1**	**hNTS2**			**ΔTemp (^**°**^C)**
NT(8-13)	H-Arg-Arg-Pro-Tyr-Ile-Leu-OH	1.5 ± 0.03	2.7 ± 0.2	0.6	1.0 ± 0.1 min	0.26 ± 0.3
1^(a)^	H-Lys-Lys-Pro-Tyr-Ile-Leu-OH	4.0 ± 0.4	1.1 ± 0.2	3.6	1.6 ± 0.3 min	0.27 ±0.2
2^(b)^	H-$\text{Lys}\psi [{\text{CH}}_{2}\text{NH}]\text{Lys}$-Pro-Tyr-Ile-Leu-OH	2.0 ± 0.8	0.31 ± 0.08	6	8.4 ± 2.0 min	−0.36 ± 0.3
3^(c)^	H-Lys-Lys-Pro-Tyr-Ile- TMSAla-OH	0.018 ± 0.004	0.25 ± 0.07	0.1	1.6 ± 0.3 min	−0.07 ± 0.1
4	H-$\text{Lys}\psi [{\text{CH}}_{2}\text{NH}]\text{Lys}$-Pro-Tyr-Ile-TMSAla-OH	2.5 ± 0.2	0.55 ± 0.1	4.5	**2.0** **±** **0.2 h**	**−2.0** **±** 0.10^****^
5^(c)^	H-Lys-Lys-Sip-Tyr-Ile-Leu-OH	14 ± 11	21 ± 4	0.7	4.5 ± 0.8 min	−0.07 ± 0.2
6	H-$\text{Lys}\psi [{\text{CH}}_{2}\text{NH}]\text{Lys}$-Sip-Tyr-Ile-Leu-OH	300 ± 50	130 ± 30	2.3	**22** **±** **2 h**	**−2.2** **±** 0.3^****^
7	H-Lys-Lys-Sip-Tyr-Ile-TMSAla-OH	55 ± 5	16 ± 4	3.4	3.5 ± 0.1 min	−0.30 ± 0.6
8	H-$\text{Lys}\psi [{\text{CH}}_{2}\text{NH}]\text{Lys}$-Sip-Tyr-Ile-TMSAla-OH	610 ± 30	24 ± 5	25	**20** **±** **4 h**	−0.22 ± 0.2
9^(a)^	H-Lys-Lys-Pro-Lys-Ile-Leu-OH	7 600 ± 1 000	310 ± 100	25	2.9 ± 0.2 min	0.32 ± 0.2
10	H- $\text{Lys}\psi [{\text{CH}}_{2}\text{NH}]\text{Lys}$-Pro-Lys-Ile-Leu-OH	6 600 ± 2 000	26 ± 15	254	**5.0** **±** **0.2 h**	0.28 ± 0.2
11^(a)^	H-Lys-Lys-Pro-Lys-Ile-TMSAla-OH	710 ± 100	76 ± 20	9.3	2.8 ± 0.1 min	0.04 ± 0.2
12	H-$\text{Lys}\psi [{\text{CH}}_{2}\text{NH}]\text{Lys}$-Pro-Lys-Ile-TMSAla-OH	150 ± 60	1.5 ± 0.7	100	**10** **±** **1 h**	**−0.41** **±** 0.2*
13	H-Lys-Lys-Pro-(D)Trp-Ile-TMSAla -OH	3 600 ± 600	8.5 ± 2	423	10 ± 2 min	0.40 ± 0.2
14	H-$\text{Lys}\psi [{\text{CH}}_{2}\text{NH}]\text{Lys}$-Pro-(D)Trp-Ile-TMSAla-OH	55 ± 3	3.5 ± 0.6	16	**19** **±** **0.3 h**	**−1.8** **±** 0.2^****^
15^(d)^	H-Lys-Lys-Pro-Dmt-Tle-Leu-OH	57 ± 6	2.4 ± 1	24	4.6 ± 0.6 min	0.14 ± 0.2
16^(d)^	H-$\text{Lys}\psi [{\text{CH}}_{2}\text{NH}]\text{Lys}$-Pro-Dmt-Tle-Leu-OH	110 ± 2	1.4 ± 0.5	79	**>24 h**	**−2.0** **±** 0.3^***^

Colored residues indicate unnatural amino acids and backbone modifications. Data are expressed ± SEM. Bold values for plasma stability; half-life of more than one hour. Bold values for hypothermia; delta of body temperature decrease at 60 min is statistically significant compared to the vehicle group.

^*^p < 0.05;

*** p < 0.001;

**** p < 0.0001.

*Please note that synthesis and binding data of some of these NT(8-13) analogs have already been reported elsewhere: ^(a)^ compounds **1**, **9, 11** in Lugrin et al., 1991; Fanelli et al., 2017; ^(b)^ compound **2** in Lugrin et al., 1991; Fanelli et al., 2017; ^(c)^ compounds **3** and **5** in Doulut et al., 1992; Vivet et al., 2000; René et al., 2013; Fanelli et al., 2015 and ^(d)^ compounds **15** and **16** in Fanelli et al., 2015; Eiselt et al., 2019. For **NT(8-13)** and **3**, the plasma stability were also reported in Doulut et al., 1992; Vivet et al., 2000; René et al., 2013; Fanelli et al., 2015*.

As previously observed (Uhl et al., 1977; St-Pierre et al., 1981; Granier et al., 1982; Lugrin et al., 1991; Held et al., 2013; Fanelli et al., 2017), arginine-to-lysine substitutions at positions 8 and 9 (compound **1**) or the insertion of the reduced amine bond between those two residues (**2**) had no major impact on NTS1/NTS2 receptor binding. As already reported (Doulut et al., 1992; Vivet et al., 2000; René et al., 2013; Fanelli et al., 2015), insertion of the TMSAla residue in position 13 (**3**) was well-tolerated by both receptors, with significantly improved affinity at NTS1 (*K*_i_ = 0.018 nM), when compared to either **1** or to the native NT(8-13) peptide (220- and 80-fold increase, respectively). However, the combination of the reduced amine bond with the TMSAla residue (**4**) induced an important loss of affinity at the NTS1 site, as compared to **3** (140-fold decrease).

Replacement of proline in position 10 by the unnatural amino acid surrogate silaproline (**5**) confers similar conformational properties to the NT(8-13) peptide. However, the presence of a dimethylsilyl group exerts protective effect against enzymatic degradation (Cavelier et al., 2002; Fanelli et al., 2015). Compared to its NT(8-13) counterpart (i.e., **1**), incorporation of Sip^10^ (**5**) decreased the affinity to both NT receptors, as previously published (Doulut et al., 1992; Vivet et al., 2000; René et al., 2013; Fanelli et al., 2015). Introduction of the hydrolytically stable CH_2_NH bond (**6**) was also found to substantially amplify the reduction in binding affinities compared to NT(8-13), with a 200- and almost 50-fold decrease in NTS1 and NTS2 binding, respectively (Table 2). In contrast, the combination of Sip with TMSAla at positions 10 and 13 (**7**) seems to have a stronger influence on NTS1 binding [35-fold decrease compared to NT(8-13)], with only a 6-fold reduction in the binding profile to NTS2. Addition of the reduced amine bond to this double substitution to produce analog **8** seems to be detrimental for NTS1, with a 400-fold decrease in affinity and no apparent change in the binding to NTS2.

We and others have previously demonstrated that Tyr^11^ is a critical position for NTS1/NTS2 affinity and selectivity (Richelson et al., 2008; Einsiedel et al., 2011; Fanelli et al., 2017; Magafa et al., 2019). As, expected, insertion of Lys at position 11 (**9**) resulted in an important loss of affinity on NTS1 and NTS2, of more than 5,000-fold and 110-fold, respectively, compared to NT(8-13). The incorporation of the reduced amine bond (**10**) had a relatively low impact on NTS1 binding affinity (*K*_i_ = 6,600 nM). However, compared to **9**, combining the reduced bond at position 8–9 with a positively charged amino acid resulted in a substantial improvement of affinity for NTS2 (*K*_i_ = 26 nM). Therefore, this combination of modifications significantly enhanced the selectivity toward NTS2 (> 250-fold) (Table 2). Incorporation of TMSAla at position 13 in presence of Lys^11^ (**11**) further increased the affinity toward both NTS1 (10-fold) and NTS2 (4-fold), compared to **9**. Likewise, the addition of the reduced Lys^8^-Lys^9^ bond, leading to compound **12** was found to be beneficial for NTS1 (4-fold) and NTS2 (50-fold) binding affinities, compared to **11**. These binding data are in accordance with previous findings (Lugrin et al., 1991; Fanelli et al., 2017).

Tyr^11^ was also replaced by two different non-natural amino acids, such as D-Trp and the tyrosine analog Dmt. Interestingly, the presence of a D-Trp in combination with a TMSAla^13^ (**13**) caused a dramatic loss in affinity for NTS1 (200,000-fold), when compared to **3**, but maintained a relatively good binding affinity to NTS2 (*K*_i_ = 8.5 nM). Then, the presence of the reduced amine bond combined with D-Trp^11^ and TMSAla^13^ (**14**) favored the binding to NTS1 by 65-fold without modifying binding at NTS2 (Table 2). Finally, as shown previously (Lugrin et al., 1991; Fanelli et al., 2017; Eiselt et al., 2019), NT(8-13) analogs harboring a Tyr mimic at position 11 (i.e., Dmt^11^) and a Tle residue at position 12, coupled or not to a CH_2_NH reduced amine bond (**15** and **16**) showed lower binding affinity at NTS1 with no significant influence on NTS2 binding.

#### Influence of the Different Proteolytic Cleavage Sites of NT(8-13) on Plasma Stability

**Figure 2:**
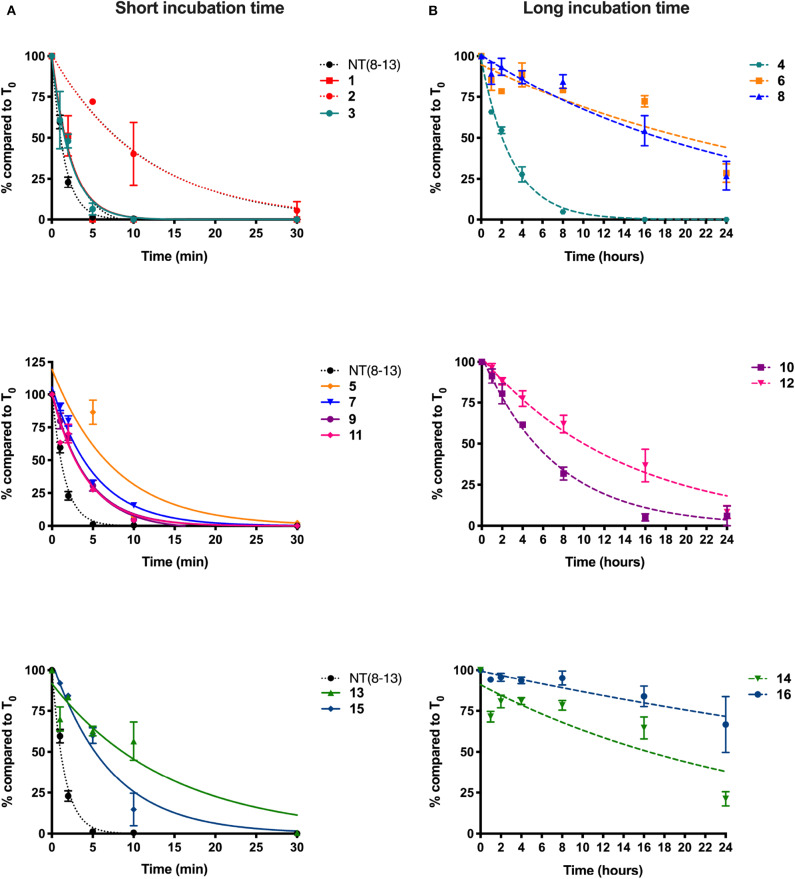
Plasma stability of novel derivatives of NT(8-13). Compounds without reduced Lys^8^-Lys^9^ pseudopeptide bond were tested for short incubation times **(A)**, while NT(8-13) analogs carrying reduced amine bonds were evaluated during longer time periods **(B)**. Determination of the percentage (%) of compounds at different incubation times in rat plasma. Half-life was calculated for each chemically modified peptide. Error bars represent mean ± SEM of at least three separate experiments for each compound.

One of the major hurdles to the use of peptides as drug candidates is their poor metabolic stability *in vivo*. As such, NT(8-13) activity is hampered by its extremely short half-life (<2 min) due to its rapid degradation *in vivo* by several endopeptidases (Fanelli et al., 2015). Enzymatic degradation of NT(8-13) occurs at three cleavage sites: Arg^8^-Arg^9^, Pro^10^-Tyr^11^, and Tyr^11^-Ile^12^ (Chart 1) (Checler et al., 1984, 1985, 1986). Here, we used different chemical strategies to stabilize the backbone of NT(8-13) in the proximity of the three cleavage sites. The stability was determined by incubating these NT(8-13) analogs for 24 h in rat plasma at 37°C (Figure 2). The half-lives of these compounds are summarized in Table 2.

In accordance with the literature (Doulut et al., 1992; Vivet et al., 2000; René et al., 2013; Fanelli et al., 2015), we observed that NT(8-13) is rapidly degraded in plasma with a half-life of 1 ± 0.1 min. As predicted, arginine-to-lysine substitutions at positions 8 and 9 (**1**) did not improve the metabolic stability. Protection of the peptide bond between the two N-terminal basic residues was found to slightly improve the half-life of **2** (T_1/2_: 8.4 ± 2.0 min). These results support previous findings demonstrating that the NT(9-13) fragment is the first metabolite of NT(8-13) (Held et al., 2013). Compound **3** modified at its C-terminal by incorporation of a TMSAla residue in position 13 exhibited similar stability than **1** (T_1/2_: 1.6 ± 0.3 min), as previously observed (Doulut et al., 1992; Vivet et al., 2000; René et al., 2013; Fanelli et al., 2015). However, the combination of TMSAla^13^ with the reduced amine bond at Lys^8^-Lys^9^ (**4**) significantly inhibited the proteolytic degradation (T_1/2_: 2.0 ± 0.2 h), thus indicating that TMSAla^13^ may limit the cleavage of the peptide bonds on the C-terminal side.

We next investigated the effects of incorporating non-natural amino acids at the two other cleavage sites, Pro^10^-Tyr^11^ and Tyr^11^-Ile^12^, on the peptide stability in plasma. To this end, Pro^10^, Tyr^11^, and Ile^12^ were, respectively, substituted by Sip, D-Trp or Dmt, and Tle. We first observed that the presence of Sip (**5**) alone or in combination with TMSAla^13^ (**7**) did not produce a protective effect on the peptide (T_1/2_ ≃ 4 min). Likewise, the di-substitution of D-Trp^11^ with TMSAla^13^ (**13**) and the presence of a Dmt-Tle in position 11-12 (**15**) did not generate NT(8-13) analogs exhibiting higher resistance to proteases (<10 min). The replacement of Tyr^11^ by Lys^11^ in presence (**11**) or not (**9**) of TMSAla^13^ was also ineffective in producing stable NT(8-13) derivatives (T_1/2_ ≃ 3 min). Interestingly, the combination of the reduced amine bond with either Sip^10^ (**6**) or Lys^11^ (**10**) led to a significant improvement in proteolytic stability (T_1/2_ = 22 and 5 h, respectively). Accordingly, addition of the reduced amine bond to the double substitution Sip^10^ and TMSAla^13^ (**8**), Lys^11^ and TMSAla^13^ (**12**), D-Trp^11^ and TMSAla^13^ (**14**), or Dmt^11^ and Tle^12^ (**16**) further increased the peptide stability, with half-lives exceeding 20 h.

#### Effect of the Peptide Backbone Modifications on NTS1-Mediated Hypothermia

**Figure 3:**
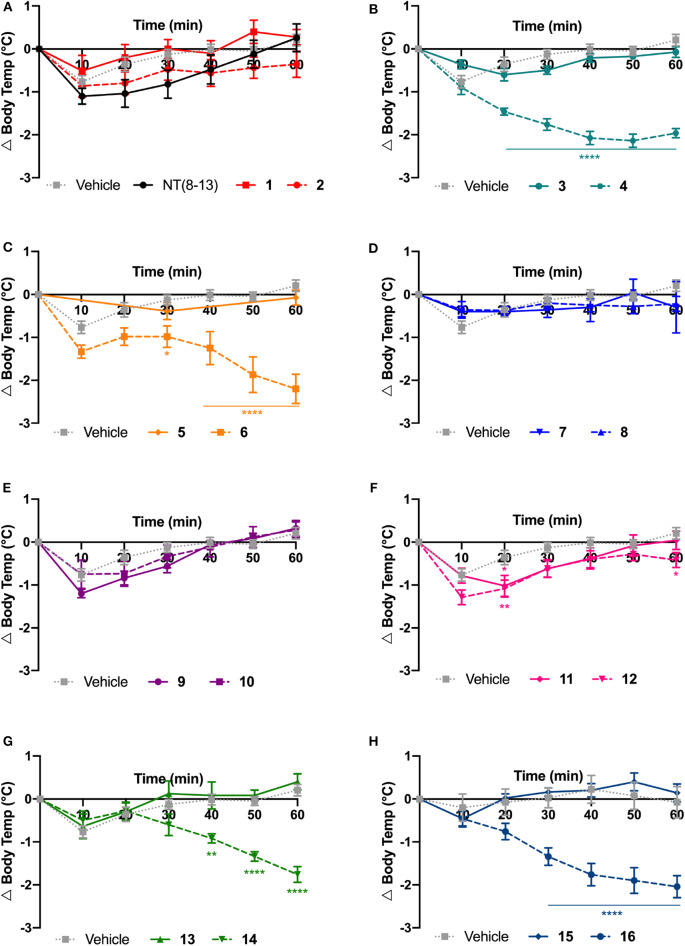
Physiological effects of NT(8-13) analogs on body temperature. Change in body temperature (Δ Body temp) was calculated at 10 min intervals over 1 h following their intrathecal injection at 30 μg/kg **(A-H)**. For each specific pair, compounds with (dotted line) or without (solid line) reduced amide bounds were represented on the same graph (*n* = 5 to 20 rats per group). Error bars represent mean ± SEM. A two-way ANOVA followed by Bonferroni's multiple comparisons test was performed. *p < 0.05; **p < 0.01; ****p < 0.0001; as compared to vehicle-injected rats.

There is now growing evidence demonstrating that NT and its derivatives induce persistent hypothermia through activation of the NTS1 receptor subtype (Liu et al., 2016). The abilities of each NT(8-13) analog to reduce body temperature were then assessed every 10 min for up to 1 h following central administration. All compounds shown in Figure 3 were injected intrathecally (i.t.) at 30 μg/kg and the change in body temperature reported in Table 2.

Spinal delivery of the chemically modified NT(8-13) analogs exhibiting high-affinity for NTS1 but poor plasma stability (i.e., **1**, **2**, **3**, **5**, **7**, and **15**) were not effective in reducing the body temperature, compared to the saline-treated group. *A fortiori*, compounds **9**, **11**, and **13** having low affinity for NTS1 and high susceptibility to protease degradation did not induce hypothermia. In sharp contrast, the highly stable compounds **4**, **14**, and **16** showed good affinity for NTS1, inducing strong and persistent hypothermia, with a temperature drop of more than 2°C for up to 60 min. Interestingly, **6** and **12** showing high resistance to protease degradation but lower affinity at NTS1 were still able to produce a slight decrease in core body temperature. Finally, the highly stable compounds **8** and **10** presenting high affinity and selectivity for NTS2 did not cause drop in body temperature, thus reinforcing the main role played by NTS1 in NT-induced hypothermia. Altogether, these results highlighted the importance of these backbone modifications in NTS1-mediating body temperature control.

## Discussion

**Figure 4:**
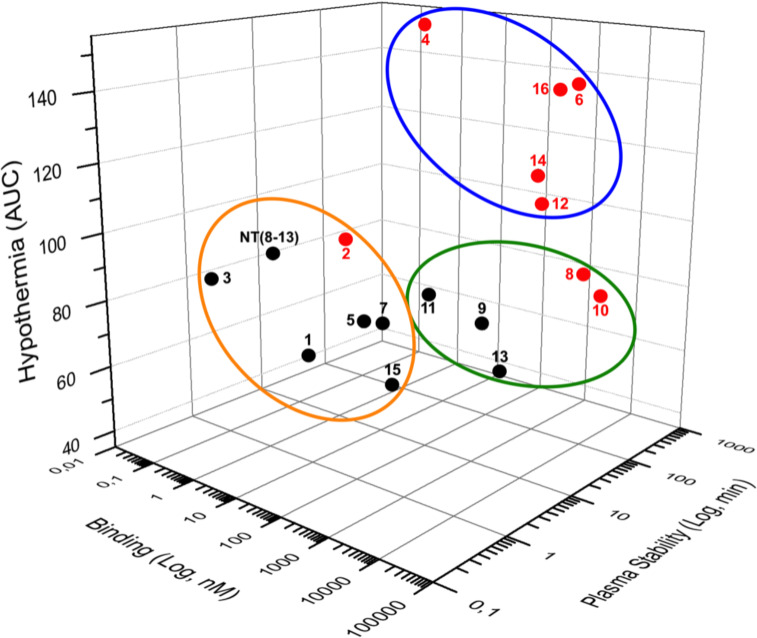
Three-dimensional (3D) data representation. Binding on NTS1, plasma stability and hypothermia represent the three coordinates of each compound. For hypothermia, results were presented as area under the curve (AUC). Compounds carrying the reduced Lys^8^-Lys^9^ bond are in red whereas non-reduced analogs are shown in black. The stable NT(8-13) analogs exhibiting a good affinity for NTS1 and inducing hypothermia are surrounded by a blue circle. The orange circle includes compounds with a good affinity for hNTS1 and short plasma half-life that do not affect body temperature. Compounds with a low affinity toward hNTS1 exerting no hypothermic action are represented with the green circle.

The extremely short half-life of NT represents a serious limitation for its potential use in pharmacological therapy. Therefore, the development of NT(8-13) analogs showing increased resistance to enzymatic cleavages of peptide bonds, while improving their biological activity *in vivo*, is of great importance. In this work, we present a panel of novel linear NT hexapeptides in which several backbone modifications, including D-amino acid or unnatural amino acid substitutions and incorporation of reduced peptide bonds were combined to stabilize the peptide bonds and increase their metabolic stability. To this aim, we synthesized a series of 8 specific pairs of NT(8-13) analogs harboring or not a reduced Lys^8^-Lys^9^ pseudopeptide bond and evaluated the effects of these chemical modifications on receptor binding, peptide stability and *in vivo* efficacy. As illustrated in Figure 4, our results demonstrated that NTS1-mediating hypothermia is mainly driven by the increased stability of the NT(8-13) derivatives, instead of the high binding affinity at NTS1.

In terms of binding, we first found that the reduced pseudopeptide bond inserted at position 8-9 (**2**) was well-tolerated. This is consistent with previous findings showing that NT(8-13) derivatives bearing a CH_2_NH bond at position 8-9 retained the receptor binding and full biological activity whereas the pseudopeptide analogs with reduced bonds at positions 10-11, 11-12, or 12-13 exhibited a marked decrease in binding affinity, in the range of two to four orders of magnitude (Lugrin et al., 1991; Wustrow et al., 1995). The importance of N-terminal positively charged amino acids for proper NTS1 binding is also in good agreement with the crystal structure of the NTS1–NT(8-13) complex in its activated-like conformation (White et al., 2012; Krumm et al., 2016). There is indeed charge complementarity between the electronegative rim of the wide open binding pocket on the extracellular surface of NTS1 and the positive-charged arginine side chains of NT(8-13). Substitution of Leu^13^ by the unnatural silicon-containing and hydrophobic amino acid TMSAla also improved the binding affinity at both NTS1 and NTS2 (**3**), giving rise to a NTS1 analog with one of the highest affinities described to date (*K*_i_ ≃ 20 pM) (Fanelli et al., 2015). Accordingly, the C-terminal end of NT(8-13) establishes hydrophobic contact with the concave NTS1 binding pocket and the negatively charged carboxylate of Leu^13^ located in an electropositive environment is predicted to form a salt bridge with Arg^327^ of NTS1 (White et al., 2012; Krumm et al., 2016). An alanine scan strategy, consisting to replace each amino acid of NT(8-13) with alanine, further supports the functional importance of the isopropyl group of Leu^13^ in receptor binding (Henry et al., 1993). The proline at position 10, playing a crucial role for peptide conformation was also replaced by the silylated proline surrogate (Sip), which displays very similar conformational properties that the proline residue (Rémond et al., 2016, 2017). The substitution of the γ-carbon by a dimethylsilyl group was found to slightly decrease the binding affinity of **5** at both NT receptors. The tightly oriented hydrophobic binding site for the pyrrolidine ring of Pro^10^, as well as the extensive van der Waals interactions with the residue Trp^339^ in ECL3 of NTS1, might in part explain this loss in receptor binding (White et al., 2012). Importantly, we also found that the NT(8-13) analogs harboring a CH_2_NH bond at position 8-9 combined to TMSAla^13^ (**4**), Sip^10^ (**6**), or to both silylated amino acids (**8**) exhibited low binding at NTS1, while the NTS2 was less affected by these chemical modifications. This is also true for compound **7**, carrying the double substitution Sip^10^ with TMSAla^13^.

Additional backbone modifications were also introduced at the Tyr^11^-Ile^12^ peptide bond. Substitution of Tyr^11^ by the lysine residue (**9**, **10**) was found to be detrimental for NT(8-13) binding at NTS1, thus leading to a substantial selectivity toward NTS2 (>250-fold). Accordingly, we recently demonstrated using molecular dynamics simulations that the Tyr^11^ residue of NT(8-13) is facing a basic residue (Arg^212^) located in the ECL2 of NTS1 or an acid residue (Glu^179^) at the same position in NTS2 (Fanelli et al., 2017). As a result, Lys^11^ of NT(8-13) is only able to form the expected salt bridge with Glu^179^ in the NTS2 receptor. As predicted, TMSAla addition to **9** and **10** (i.e., **11** and **12**, respectively) led to a substantial improvement in binding affinity at both NTS1 and NTS2. The importance of the tyrosine residue in position 11 was further investigated by replacing Tyr^11^ by D-Trp^11^. We found that the incorporation of a bulky aromatic D-isomer combined to TMSAla^13^ (**13**) caused a dramatic loss of binding affinity at NTS1, when compared to **3** (200,000-fold) and subsequent NTS2 selectivity (>420-fold). This result is consistent with recent findings indicating that Tyr^11^ is a key residue for determining receptor selectivity, extension of the aromaticity, and changes in side chain orientation, favoring selectivity toward the NTS2 receptor (Dubuc et al., 1999; Richard et al., 2001; Boules et al., 2010; Einsiedel et al., 2011; Held et al., 2013; Fanelli et al., 2017; Eiselt et al., 2019). Strikingly, addition of the reduced Lys-Lys bond to **13** greatly restored the binding affinity at NTS1 (**14**). Despite the fact that peptides containing <20 amino acid residues lack significant secondary structure, they can adopt specific bioactive conformations to interact with the biological target. In this context, we might hypothesize that the multiple modifications introduced into the NT(8-13) peptide can dramatically modify the peptide structure and thereby alter its ability to bind to NTS1. Finally, NT(8-13) analogs harboring Dmt^11^-Tle^12^, coupled or not to a CH_2_NH reduced amine bond (**15**, **16**), showed lower binding affinity at NTS1 than NTS2, as expected by the importance of position 11 in receptor subtype selectivity. Altogether, these results demonstrated that NTS2 is more permissive than NTS1 to peptide backbone substitutions. Similarly, with a few exceptions, analogs bearing the reduced moiety showed better affinity at NTS2 when compared to the corresponding non-reduced analogs. These findings are well-supported by the high-resolution crystal structure of NTS1, which reveals that the overall shape of the NTS1 ligand-binding pocket is narrower than that observed in other peptide receptors (White et al., 2012).

In the present study, we further evaluated different strategies for improving proteolytic resistance of NT(8-13) peptide analogs. We found that only the combination of appropriate chemical modifications led to the development of metabolically stable analogs. Indeed, compounds harboring only the reduced Lys-Lys bond (**2**) or a single amino substitution (**3**, **5**, and **9**) exhibited extremely short half-life (<10 min). Likewise, the di-substituted compounds Sip^10^ - TMSAla^13^ (**7**), Lys^11^ - TMSAla^13^ (**11**), D-Trp^11^ - TMSAla^13^ (**13**), and Dmt^11^ - Tle^12^ (**15**) generated to limit the enzymatic cleavages of the Pro^10^-Tyr^11^ and Tyr^11^-Ile^12^ bond sites were not stable in rat plasma (<10 min), leaving the N-terminus accessible to the metalloendopeptidase (i.e., EC 3.4.24.15) and aminopeptidases (Checler et al., 1984, 1985, 1986; Orwig et al., 2009). Interestingly, our results revealed that the combination of the reduced Lys-Lys bond with Sip^10^ (**6**), Lys^11^ (**10**), or TMSAla^13^ (**4**) resulted in significant improvement of the peptide plasma stability (half-lives ranging from 2 to 22 h). This is quite surprising since all of these NT(8-13) derivatives are still supposed to be cleavable by the endopeptidases EC 3.4.24.11 and EC 3.4.24.16 at the Pro^10^-Tyr^11^ or Tyr^11^-Ile^12^ positions (Checler et al., 1984, 1985, 1986). This increased metabolic stability might be explained by the fact that the chemical modifications introduced in the NT(8-13) peptide can reshape the peptide structure in a conformation not suitable for its recognition by peptidases. This hypothesis is supported by previous findings showing that some chemical modifications quite distant from the susceptible site can dramatically affect the cleavage efficiency by peptidases. For instance, the growth hormone releasing factor GRF(1-29)-NH2 can be protected against its proteolytic hydrolysis in plasma by the dipeptidylpeptidase IV (DPP IV) between positions 2 and 3 by chemical modifications distal to the N-terminus, such as at the residue positions 21 and 25 (Su et al., 1991). Likewise, steric changes of the peptide structure have been proposed to explain the increased susceptibility of the cytotoxic T-lymphocyte-epitope 13 amino acid peptide to endopeptidases following terminal modifications (Marschutz et al., 2002). Finally, protection of the different proteolytic cleavage sites by the combination of the reduced amine bond with the double substitution Sip^10^-TMSAla^13^ (**8**), Lys^11^-TMSAla^13^ (**12**), D-Trp^11^-TMSAla^13^ (**14**), or Dmt^11^-Tle^12^ (**16**) abolished sufficiently the accessibility of the susceptible sites to peptidases to extend their plasma stability for at least 24 h. Overall, these results highlight the importance of the reduced amine bond in optimizing the metabolic properties of the NT(8-13) peptide and reinforce the need to combine appropriate chemical modifications to generate stable NT(8-13) analogs exhibiting prolonged plasma half-life *in vivo*.

Hypothermia therapy has been found to exert neuroprotective effects in many neurological diseases, such as stroke, traumatic brain injury, intracranial pressure elevation, and neonatal encephalopathy (Huber et al., 2019; Sun et al., 2019). In light of this, there is now growing evidence demonstrating that NT(8-13) analogs induce regulated reduction of body and brain temperatures through activation of the NTS1 receptor subtype (Liu et al., 2016). Indeed, administration of the NTS1 agonists, NT69L, PD149163, HPI-201, or HPI-363, produced mild hypothermia and improved the neurological outcomes compared with that of results from external cooling (Katz et al., 2001; Choi et al., 2012; Wei et al., 2013; Lee et al., 2014, 2016; Gu et al., 2015; Xue et al., 2017; Zhao et al., 2020; Zhong et al., 2020). Here, we therefore determined the ability of these specific pairs of NT(8-13) analogs to regulate the body temperature and evaluated the contribution of the receptor binding affinity and peptide plasma stability on the hypothermic activity. Our data clearly demonstrated that the peptide metabolic stability is more important to the hypothermic response of the NT(8-13) analogs than the receptor binding affinity. Indeed, all NT(8-13) derivatives exhibiting a high-affinity at NTS1 but low plasma stability (**1**, **2**, **3**, **5**, **7**, and **15**) were not effective to induce drop in body temperature, while highly stable compounds (**4**, **14**, and **16)** showing good affinity for NTS1 exerted long-lasting hypothermia. This latter observation was further strengthened by the presence of a hypothermic response following administration of **6** and **12**, which show a high resistance to protease degradation but a relatively low affinity at NTS1. As expected, compounds **9**, **11**, and **13** having low affinity for NTS1 and high susceptibility to peptidases did not cause drop in body temperature. Consistent with our results, Orwig et al. identified that the N-terminal capping of NT(8-13) leading to increased stability *in vivo* can compensate for a reduction in binding affinity to NTS1 (Orwig et al., 2009). Accordingly, the neuropeptide neuromedin N, a closely related hexapeptide of NT(8-13), which does not normally produce hypothermia due to its poor stability can, however, induce a hypothermic effect when administered in the presence of peptidase inhibitors (Dubuc et al., 1988).

In conclusion, these results indicate that these backbone modifications to the NT(8-13) peptide are required for NTS1-mediating hypothermia and that the relatively good binding affinity for NTS1 does not translate directly into biological responses, the main driver of this physiological effect being the increased *in vivo* stability.

## Data Availability Statement

All datasets generated for this study are included in the article/Supplementary Material.

## Ethics Statement

The animal study was reviewed and approved by the Animal Care Committee of the Université de Sherbrooke (protocol n°035-18B) and were in accordance with policies and directives of the Canadian Council on Animal Care.

## Author Contributions

SP was in charge of synthesizing the compounds and writing the chemical part of the manuscript. MV and SBe performed the biological part of the study and MV wrote the *in vitro* and *in vivo* sections. The manuscript was written by MV, SP, ER, J-ML, SBa, FC, and PS. All authors have given their approval to the final version of the manuscript.

## Conflict of Interest

The authors declare that the research was conducted in the absence of any commercial or financial relationships that could be construed as a potential conflict of interest. The handling editor declared a past co-authorship with one of the authors SBa.
