# Supplementary material for: Insightful Backbone Modifications Preventing Proteolytic Degradation of Neurotensin Analogs Improve NTS1-Induced Protective Hypothermia
Source: Front Chem. 2020 Jun 5;8:406. doi: 10.3389/fchem.2020.00406 (PMC7291367; doi:10.3389/fchem.2020.00406)
Supplement: Supplementary file 1 [file Data_Sheet_1.docx]

**Supplementary Information**

**Insightful backbone modifications preventing proteolytic degradation of neurotensin analogues improve NTS1-induced protective hypothermia**

Santo Previti^1,3,#^, Mélanie Vivancos^2,#^, Emmanuelle Rémond^1^, Sabrina Beaulieu^2^, Jean-Michel Longpré^2^, Steven Ballet^3^, Philippe Sarret^2^**^*^**, Florine Cavelier^1^**^*^**

^1^Institut des Biomolécules Max Mousseron, IBMM, UMR-5247, CNRS, Université de Montpellier, ENSCM, Place Eugène Bataillon, 34095 Montpellier cedex 5, France.

^2^Department of Pharmacology-Physiology, Faculty of Medicine and Health Sciences, Institut de Pharmacologie de Sherbrooke, Université de Sherbrooke, Sherbrooke, Québec, Canada J1H 5N4.

^3^Research Group of Organic Chemistry, Departments of Bioengineering Sciences and Chemistry, Vrije Universiteit Brussel, Pleinlaan 2, Brussels 1050, Belgium.

^#^ These authors contributed equally: Santo Previti and Mélanie Vivancos

**^*^ Co- corresponding authors:**

**Florine Cavelier, Ph.D. Philippe Sarret, Ph.D.**

Institut des Biomolécules Max Mousseron Department of Pharmacology-Physiology

IBMM, UMR-5247, CNRS Faculty of Medicine and Health Sciences

Université Montpellier, ENSCM Université de Sherbrooke

Place Eugène Bataillon 3001, 12^th^ Avenue North

34095 Montpellier cedex 5 Sherbrooke, Québec, J1H 5N4

France Canada

Tel: (+33) 467143765 Tel: (819) 821-8000, Ext: 72554

Email: [florine.cavelier@umontpellier.fr](mailto:florine.cavelier@umontpellier.fr) Email: [Philippe.Sarret@USherbrooke.ca](mailto:Philippe.Sarret@USherbrooke.ca)

**INDEX**

**Materials & methods 3**

**General procedures for peptide synthesis 4**

**Synthesis and characterization of novel NT(8-13) analogues 6**

**Tables 25**

**References 26**

**MATERIALS AND METHODS**

All solvents were purchased from Sigma Aldrich in gradient grade or reagent quality. All reactions involving air-sensitive reagents were performed under nitrogen or argon. Purifications were performed with column chromatography using silica gel (Merck 60, 230–400 mesh). LC/MS system consisted of a Waters Alliance 2690 HPLC, coupled to a ZQ spectrometer (Manchester, UK) fitted with an electrospray source operated in the positive ionization mode (ESI^+^). All the analyses were carried out using a C18 Chromolith Flash 25 x 4.6 mm column operated at a flow rate of 3 ml/min. A gradient of 0 to 100% solvent B was used over 3 min. Positive-ion electrospray mass spectra were acquired at a solvent flow rate of 100-200 µL/min. Nitrogen was used for both the nebulizing and drying gas. The data were obtained in a scan mode ranging from 200 to 1700 m/z in 0.1 s intervals. A total of 10 scans were summed up to get the final spectrum. All the fully unprotected peptides were purified using a gradient composed of water/acetonitrile with 0.1% TFA at 50 mL/min flow rate. Purity was determined by HPLC Agilent 1200 equipped with a column Onyx monolithic HD-C18 50 x 4.6 mm following a gradient of 0 to 100 % of ACN with 1/1000 of TFA in 10 minutes. All peptides were obtained with purity higher than 95%. High resolution mass spectra (HRMS) were performed at the “Laboratoire de Mesures Physiques” of Montpellier University on a Micromass Q-Tof spectrometer equipped with electrospray source ionization (ESI), using phosphoric acid as an internal standard. All the fully unprotected peptides were purified using a gradient composed of water/acetonitrile with 0.1% TFA at 50 mL/min flow rate performed on a PLC2020 Gilson® using a 75 x 21.2 mm Phenomenex® Luna 5u C18(2) column. Purity was determined by. RP-Analytic HPLC performed on a Agilent 1220 using a 50 x 4.6 mm Chromolith® High Resolution column. Compounds were separated using a linear gradient system (0 to 100% solvent B in 10 min) using a constant flow rate of 3mL/ min. High resolution mass spectra (HRMS) were performed at the “Laboratoire de Mesures Physiques” of Montpellier University on a Micromass Q-Tof spectrometer equipped with electrospray source ionization (ESI), using phosphoric acid as internal standard.

**GENERAL PROCEDURES**

Unnatural amino acids silaproline (Boc-Sip-OH) ([Vivet et al., 2000](#_ENREF_1)), trimethylsilylalanine (Boc-TMSAla-OMe) ([René et al., 2013](#_ENREF_2); Fanelli et al., 2015) and pseudopeptide Boc-Lys(Boc)ψ[CH_2_NH]Lys(Boc)-OH ([Doulut et al., 1992](#_ENREF_3)) were prepared according to reported literature procedures.

***General procedure for coupling reaction in solution (GP1)***

To a solution of the amino ester or peptide in DMF, the suitable NH protected amino acid or peptide was added followed by the addition of HATU (1.2 eq.) and N,N-diisopropyethylamine (3 eq.). The mixture was stirred at room temperature overnight. The solvent was evaporated and the residue was diluted with EtOAc and consecutively extracted with 1 M KHSO_4_ (2×), aqueous NaHCO_3_ (2×) and brine (2×), dried over Na_2_SO_4_ and the solvent evaporated under reduced pressure to afford the crude product which was then purified on silica gel and characterized by LC-MS.

***General procedure for BOC deprotection in solution with TFA (GP2)***

The protected peptide was dissolved in TFA (4 mL/mmol), in the presence of TIS (10%). The reaction was stirred 2.5 h at room temperature. Volatiles were removed under reduced pressure. The resulting residue was precipitated in diethyl ether and subsequently filtered and dried, to afford the corresponding TFA salt. The crude peptides were purified by RP-HPLC.

***General procedure for methyl ester saponification (GP3)*** The peptide methyl ester was dissolved in methanol (10 ml/mmol) and a solution of 4N KOH (5 eq.) was added. The reaction was stirred until disappearance of the starting material. The solvent was removed under reduced pressure and the resulting residue was dissolved in water (10 mL/mmol), then acidified with citric acid 15% to pH 4-5. The organic product was extracted with ethyl acetate (x 4), the organic layer was dried over MgSO_4_ and concentrated *in vacuo*, to afford the corresponding carboxylic acid, which was used for the next step without further purification.

***General procedure for Bn and Z deprotection of peptides in solution (GP4)***

The protected peptide was dissolved in methanol (5 mL/mmol) and 10% Pd/C under hydrogen atmosphere. The reaction was stirred 3 h at room temperature. The mixture was filtered on a pad of celite, which was washed with methanol. The solvent was evaporated under reduced pressure to afford the free amine.

**Synthesis of compound 4 (H-Lysψ[CH_2_NH]Lys-Pro-Tyr-Ile-TMSAla-OH)**

Compound **4** was prepared according to a 3+3 fragment coupling as depicted in Scheme S1. The C-terminal tripeptide H-Tyr(Bn)-Ile-TMSAla-OMe was synthesized following Boc-chemistry in solution, and general procedure (GP) 1 and GP2 for coupling and Boc-deprotection, respectively. Pseudopeptide Boc-Lys(Boc)ψ[CH_2_NH]Lys(Boc)-OH was prepared as reported in literature ([Doulut et al., 1992](#_ENREF_3)). The subsequent coupling H-Pro-OMe provided the tripeptide intermediate Boc-Lys(Boc)ψ[CH_2_NH]Lys(Boc)-Pro-OMe, which after saponification following GP3, gave the corresponding acid. A coupling between tripeptides Boc-Lys(Boc)ψ[CH_2_NH]Lys(Boc)-Pro-OH and H-Tyr(Bn)-Ile-TMSAla-OMe provided the desired hexapeptide carrying the protecting groups. Saponification (GP3), removal of the benzyl on the side chain of Tyr (GP4) and TFA treatment (GP2) presented the desired compound **4**. The presence of a proline residue and a reduced amide bond avoided racemization during the assembly process. The overall yield was 14% (over 10 steps). Calculated mass: 777.5059; found mass: 777.5059; HPLC retention time: 1.46 min. HPLC chromatograms and mass spectra are reported below.

**Scheme S1.** Synthesis of compound **4**. Reagents and conditions: GP1: HATU, DIPEA, DMF, rt, o.n.; GP2: TFA, 10% TIS, 2.5 h, rt; GP3: 4N KOH, MeOH, rt; GP4: H_2_, 10% Pd/C, MeOH, 3h, rt.

**Figure S1.** HPLC profile of peptide **4**.


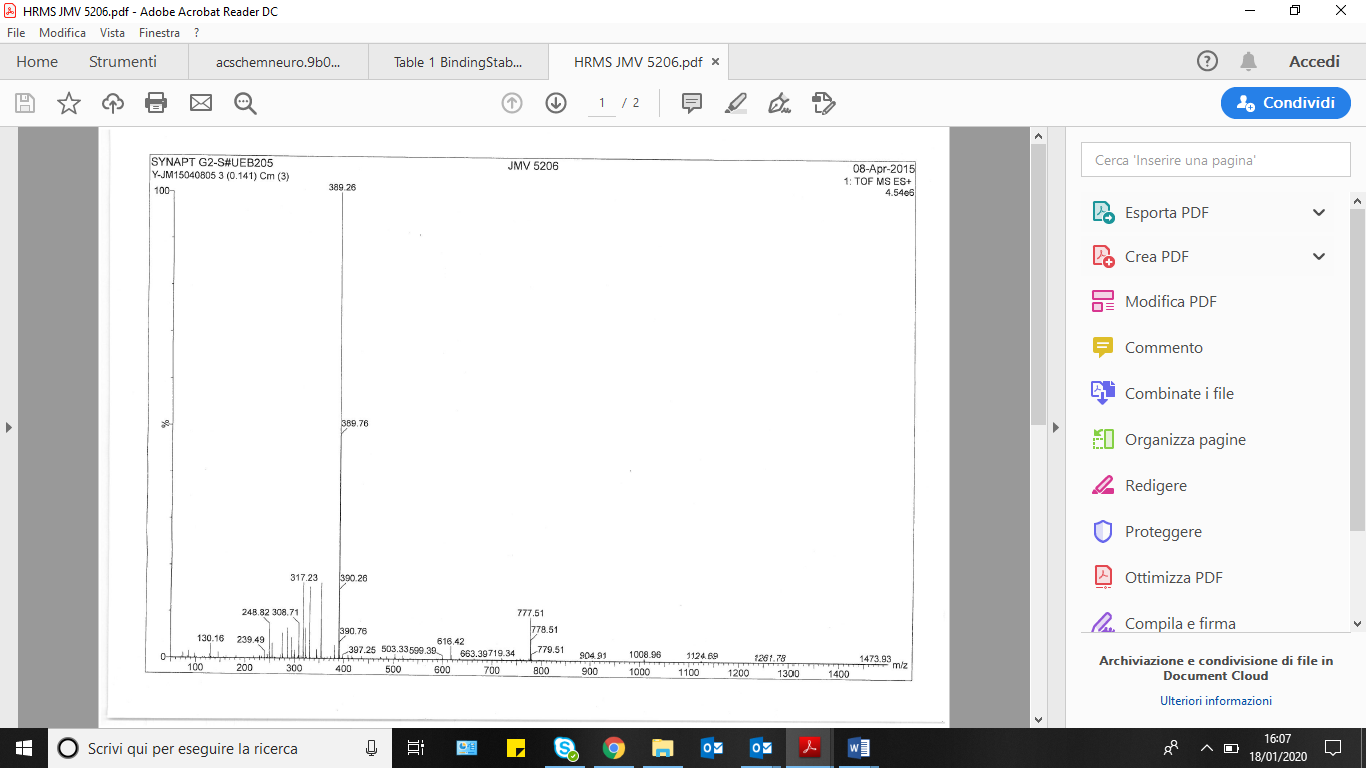


**Figure S2.** Mass spectra of peptide **4**.

**Figure S3.** High-Resolution mass spectra of peptide **4**.

**Synthesis of compound 6 (H-Lysψ[CH_2_NH]Lys-Sip-Tyr-Ile-Leu-OH)**

Compound **6** was prepared in accordance to a 2+4 fragment coupling as depicted in Scheme S2. In contrast to compound **4**, the amino acid in position 10 (Sip) was inserted into the C-terminal subsequence. The tetrapeptide H-Sip-Tyr(Bn)-Ile-Leu-OMe was coupled with the building block Boc-Lys(Boc)ψ[CH_2_NH]Lys(Boc)-OH, providing the fully protected hexapeptide Boc-Lys(Boc)ψ[CH_2_NH]Lys(Boc)-Sip-Tyr(Bn)-Ile-Leu-OMe. Subsequently, saponification (GP3), hydrogenation (GP4) and Boc-deprotection (GP2) provided the desired product. Calculated mass: 791.5225; found mass: 791.5215; LC-MS retention time: 0.85 min. Overall yield: 21%. LC-MS and mass spectra are reported below.

**Scheme S2.** Synthesis of compound **6**. Reagents and conditions. GP1: HATU, DIPEA, DMF, rt, o.n.; GP2: TFA, 10% TIS, 2.5h, rt; GP3: 4N KOH, MeOH, rt; GP4: H_2_, 10% Pd/C, MeOH, 3h, rt.

**Figure S4.** LCMS of peptide **6**.

| A) |
| --- |
| B) |
|  |

**Figure S5.** Mass spectra of peptide **6**.

**Figure S6.** High-Resolution mass spectrum of compound **6**.

**Synthesis of compound 7 (H-Lys-Lys-Sip-Tyr-Ile-TMSAla-OH)**

Compound **7** was prepared following the standard approach for peptide synthesis in solution, with a build-up from the C-terminal position 13 (H-TMSAla-OMe, Scheme S3). Unlike compound **6**, we followed the Boc-chemistry strategy for all couplings (5 in all). Calculated mass: 834.4855; found mass: 835.49; HPLC retention time over 10 min: 3.673 min; Purity > 95%. Overall yield: 17% (11 steps). The HPLC chromatogram and mass spectrum are reported below.

**Scheme S3.** Synthesis of compound **7**. Reagents and conditions. GP1: HATU, DIPEA, DMF, rt, o.n.; GP2: TFA, 10% TIS, 2.5h, rt; GP3: 4N KOH, MeOH, rt; GP4: H_2_, 10% Pd/C, MeOH, 3h, rt.

**Figure S7.** HPLC profile of hexapeptide **7**.

**Figure S8.** Mass spectrum of compound **7**

**Synthesis of compound 8 (H-Lysψ[CH_2_NH]Lys-Sip-Tyr-Ile-TMSAla-OH)**

Compound **8** was prepared following the procedure above for compound **6**, and using a 2+4 fragment coupling as depicted in Scheme S4, by coupling Boc-Lys(Boc)ψ[CH_2_NH]Lys(Boc)-OH with H-Sip-Tyr(Bn)-Ile-TMSAla-OMe. The presence of reduced amide bond in the dipeptide Boc-Lys(Boc)ψ[CH_2_NH]Lys(Boc)-OH avoided racemization process. Overall yield: 22% (10 steps); Calculated mass (M/2): 411.2579; found mass (M/2): 411.3; Mass spectra are reported below.

**Scheme S4.** Synthesis of compound **8**. Reagents and conditions. GP1: HATU, DIPEA, DMF, rt, o.n.; GP2: TFA, 10% TIS, 2.5h, rt; GP3 : 4N KOH, MeOH, rt; GP4: H_2_, 10% Pd/C, MeOH, 3h, rt.

| A) |
| --- |
| B) |

**Figure S9.** Mass spectra of compound **8**.

**Synthesis of 10 (H-Lysψ[CH_2_NH]Lys-Pro-Lys-Ile-Leu-OH)**

Compound **10** was prepared in according to the procedure described for compound **4,** by coupling Boc-Lys(Boc)ψ[CH_2_NH]Lys(Boc)-Pro-OH and H-Lys(Z)-Ile-Leu-OMe (Scheme 5). Also in this case, the presence of proline in the tripeptide Boc-Lys(Boc)ψ[CH_2_NH]Lys(Boc)-Pro-OH avoided the racemization process. Overall yield: 16% (10 steps; purity > 95%). Calculated mass: 712.5450; found mass: 712.5; LC-MS retention time: 0.63 min. The LC-MS analysis is reported below.

**Scheme S5.** Synthesis of compound **10**. Reagents and conditions. GP1: HATU, DIPEA, DMF, rt, o.n.; GP2: TFA, 10% TIS, 2.5h, rt; GP3: 4N KOH, MeOH, rt; GP4: H_2_, 10% Pd/C, MeOH, 3h, rt.

| A)   |
| --- |
| B)   |

**Figure S10.** LC (A) and mass spectrum (B) of peptide **10**.

**Synthesis of compound 12 (H-Lysψ[CH_2_NH]Lys-Pro-Lys-Ile-TMSAla-OH)**

Compound **12** was prepared following the procedure above for compound **4**, by coupling Boc-Lys(Boc)ψ[CH_2_NH]Lys(Boc)-Pro-OH and H-Lys(Z)-Ile-TMSAla-OMe (Scheme S6). The racemization process was avoided due to the presence of reduced amide bond (*i.e.* coupling between Boc-Lys(Boc)ψ[CH_2_NH]Lys(Boc)-OH and H-Pro-OMe) and proline (3 + 3 coupling). Overall yield: 14% (10 steps). Calculated mass: 742.5376; found mass: 742.5; LC-MS retention time: 0.64 min; Purity > 95%. LC-MS is reported below.

**Scheme S6.** Synthesis of compound **12**. Reagents and conditions. GP1: HATU, DIPEA, DMF, rt, o.n.; GP2: TFA, 10% TIS, 2.5h, rt; GP3: 4N KOH, MeOH, rt; GP4: H_2_, 10% Pd/C, MeOH, 3h, rt.

| A)   |
| --- |
| B)  **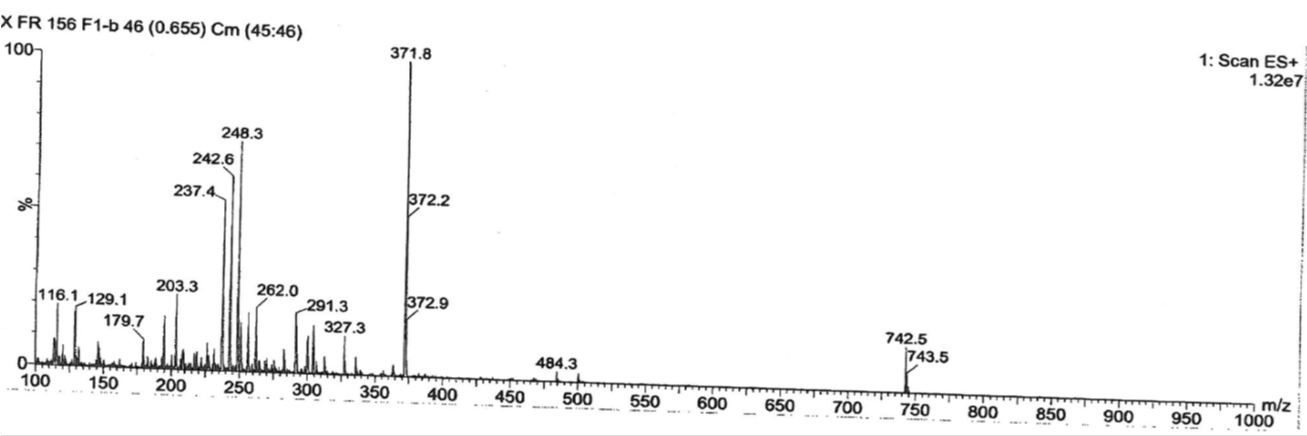** |

**Figure S11.** LC-MS analysis of compound **12**.

**Synthesis of compound 13 (H-Lys-Lys-Pro-D-Trp-Ile-TMSAla-OH)**

Compound **13** was prepared according to a 3+3 fragment coupling as shown in Scheme S7. As it can be noted, D-Trp was used without any protecting group on the side chain: in order to avoid undesirable side products, we limited the number of reactions in which D-Trp was included. For this reason, tripeptide H-D-Trp-Ile-TMSAla-OMe was coupled with the acid tripeptide Boc-Lys(Boc)-Lys(Boc)-Pro-OH, and only a very low percentage of side products were detected. Presence of proline in position 10 avoided racemization. Overall yield: 11% (12 steps). Calculated mass (M/2): 407.7545; found mass (M/2): 407.7; HPLC retention time over 5 min: 1.60 min; Purity > 95%. The HPLC chromatogram and mass spectra are reported below.

**Scheme S7.** Synthesis of compound **13**. Reagents and conditions. GP1: HATU, DIPEA, DMF, rt, o.n.; GP2: TFA, 10% TIS, 2.5h, rt; GP3: 4N KOH, MeOH, rt.

**Figure 12S**. HPLC profile of hexapeptide **13**.

|  |
| --- |
|  |

**Figure 13S.** Mass spectra of compound **13**.

**Synthesis of compound 14 (H-Lysψ[CH_2_NH]Lys-Pro-D-Trp-Ile-TMSAla-OH)**

Compound **14** was synthesized as described for compound **13**, by coupling Boc-Lys(Boc)ψ[CH_2_NH]Lys(Boc)-Pro-OH with H-D-Trp-Ile-TMSAla-OMe (Scheme S8). Overall yield: 10% (9 steps). Calculated mass (M/2): 400.7649; found mass (M/2): 400.7661. Mass spectra are reported below.

**Scheme S8.** Synthesis of compound **14**. Reagents and conditions. GP1: HATU, DIPEA, DMF, rt, o.n.; GP2: TFA, 10% TIS, 2.5h, rt; GP3: 4N KOH, MeOH, rt.

|  |
| --- |
|  |

**Figure 14S**. Mass spectra of compound **14**.

**Table S1.** References for each compound already reported in literature.

| **Cmp** | **Sequence** | **Reference** | **Code in the reference paper** |
| --- | --- | --- | --- |
| **1** | H-Lys-Lys-Pro-Tyr-Ile-Leu-OH | ([Fanelli et al., 2017](#_ENREF_4)) | 1 |
| **2** | H-LysΨ[CH_2_NH]Lys-Pro-Tyr-Ile-Leu-OH | ([Lugrin et al., 1991](#_ENREF_5)) | H-[*Ψ*8,9] |
| **3** | H-Lys-Lys-Pro-Tyr-Ile-TMSAla-OH | ([Fanelli et al., 2015](#_ENREF_6)) | 6 |
| **5** | H-Lys-Lys-Sip-Tyr-Ile-Leu-OH |  | 10 |
| **9** | H-Lys-Lys-Pro-Lys-Ile-Leu-OH | ([Fanelli et al., 2017](#_ENREF_4)) | 2 |
| **11** | H-Lys-Lys-Pro-Lys-Ile-TMSAla-OH |  | 7 |
| **15** | H-Lys-Lys-Pro-Dmt-Tle-Leu-OH | ([Eiselt et al., 2019](#_ENREF_7)) | 5 |
| **16** | H-LysΨ(CH_2_NH)Lys-Pro-Dmt-Tle-Leu-OH |  | 9 |

**Table S2.** Summary data for the new synthesized peptides.

| **Cmp** | Calculated mass | Signal in mass spectrometry | Retention time |
| --- | --- | --- | --- |
| **4** | 777.5059 | 777.5059 | 1.46 min  HPLC over 5 min |
| **6** | 791.5225 | 791.5215 | 0.85 min  LCMS over 3 min |
| **7** | 834.4855 | 835.49 | 3.67 min  HPLC over 10 min |
| **8** | 411.2579 (M/2) | 411.3  (M/2) | Not available |
| **10** | 712.5450 | 712.5 | 0.63 min  LCMS over 3 min |
| **12** | 742.5376 | 742.5 | 0.64 min  LCMS over 3 min |
| **13** | 407.7545 | 407.7 | 1.60 min  HPLC over 5 min |
| **14** | 400.7649 | 400.7661 | Not available |

**REFERENCES**

Doulut, S., Rodriguez, M., Lugrin, D., Vecchini, F., Kitabgi, P., Aumelas, A. and Martinez, J. (1992). Reduced peptide bond pseudopeptide analogues of neurotensin. *Pept Res* 5, 30-38.

Eiselt, E., Gonzalez, S., Martin, C., Chartier, M., Betti, C., Longpre, J. M., Cavelier, F., Tourwe, D., Gendron, L., Ballet, S. and Sarret, P. (2019). Neurotensin analogues containing cyclic surrogates of tyrosine at position 11 improve NTS2 selectivity leading to analgesia without hypotension and hypothermia. *ACS Chem Neurosci* 10, 4535-4544. doi: 10.1021/acschemneuro.9b00390

Fanelli, R., Besserer-Offroy, É., René, A., Côté, J., Tétreault, P., Collerette-Tremblay, J., Longpré, J.-M., Leduc, R., Martinez, J., Sarret, P. and Cavelier, F. (2015). Synthesis and characterization in vitro and in vivo of (L)-(trimethylsilyl)alanine containing neurotensin analogues. *J Med Chem* 58, 7785-7795. doi: 10.1021/acs.jmedchem.5b00841

Fanelli, R., Floquet, N., Besserer-Offroy, E., Delort, B., Vivancos, M., Longpré, J. M., Renault, P., Martinez, J., Sarret, P. and Cavelier, F. (2017). Use of molecular modeling to design selective NTS2 neurotensin analogues. *J Med Chem* 60, 3303-3313. doi: 10.1021/acs.jmedchem.6b01848

Lugrin, D., Vecchini, F., Doulut, S., Rodriguez, M., Martinez, J. and Kitabgi, P. (1991). Reduced peptide bond pseudopeptide analogues of neurotensin: binding and biological activities, and in vitro metabolic stability. *Eur J Pharmacol* 205, 191-198. doi: 10.1016/0014-2999(91)90819-c

René, A., Vanthuyne, N., Martinez, J. and Cavelier, F. (2013). (L)-(Trimethylsilyl)alanine synthesis exploiting hydroxypinanone-induced diastereoselective alkylation. *Amino Acids* 45, 301-307. doi: 10.1007/s00726-013-1492-2

Vivet, B., Cavelier, F. and Martinez, J. (2000). Synthesis of Silaproline, a New Proline Surrogate. *Eur J Org Chem* 2000, 807-811. doi: 10.1002/(SICI)1099- 0690(200003)2000:5<807::AID-EJOC807>3.0.CO;2-E
